# Radiographic outcome of surgical treatment of adolescent idiopathic scoliosis in males versus females

**DOI:** 10.1186/1748-7161-3-12

**Published:** 2008-09-06

**Authors:** Ebrahim Ameri, Hamid Behtash, Bahram Mobini, Farzad Omidi-Kashani, Behnam Momeni

**Affiliations:** 1 Department of Spine Surgery, Shafa Yahyaiian Hospital, Iran University of Medical Sciences, Tehran, Iran; 2 Department of Orthopedic Surgery, Shafa Yahyaiian Hospital, Iran University of Medical Sciences, Tehran, Iran

## Abstract

**Background:**

Studies on adolescent idiopathic scoliosis have well documented the differences between natural history of male and female patients. There are also differences in responses to nonoperative treatment, but the results of operative treatment in male patients compared with females have not been widely reported. Only few studies had compared the outcomes of operative treatment between male and female patients with different results.

**Methods:**

We retrospectively reviewed the outcome of 150 (112 girls and 38 boys) consecutive patients with diagnosis of adolescent idiopathic scoliosis who were managed surgically between May 1996 and September 2005. Next, male radiographic parameters were compared with female ones pre- and postoperatively. Then, a subgroup of 38 matched girls was compared regarding the age, curve type, curve magnitude, and the instrumentation we used.

**Results:**

In comparing male patients with unmatched girls, the boys had greater mean age (17.3 ± 2.3 vs. 16.3 ± 2.9; p = 0.049), greater primary curve (71.4 ± 21.3° vs. 62.7 ± 17.5°; p = 0.013), less flexibility (30.1 ± 13.5% vs. 40.3 ± 17.8%; p = 0.01), and less correction percentage (51.3 ± 12.9% vs. 58.8 ± 16.5%; p = 0/013). The loss of correction was comparable between the two groups. In the matched comparison, the flexibility in boys was less than girls (30.1 ± 13.5% vs. 38.1 ± 17.5%; p = 0.027). Also, the boys had a smaller correction percentage compared to the girls, but this finding was not statistically significant.

**Conclusion:**

There was similar distribution curve pattern between male and female patients with AIS. Males had more rigid primary curves compared to females but a similar degree of postoperative scoliosis correction. Male AIS patients were older at the time of surgery. These preoperative gender differences, however; did not compromise the radiological outcomes of surgical treatment and the results were comparable between the genders.

## Background

Adolescent idiopathic scoliosis (AIS) is a structural three-dimensional deformity of the spine that occurs at or near the onset of puberty for which no cause can be established. In patients with small curve magnitude in the mean of 10° or so, the male and female prevalence is approximately equal. In curves of larger magnitude, however, there is an overwhelming female predominance in a way that the ratio of females to males with curves measuring 30° or more is 10 to 1 [1,2].

According to epidemiologic and natural history studies, curve progression is different in male and female patients. Studies conducted by Suh and MacEwen [3], and Karol et al [4] on curve behavior in males verified that scoliotic male patients demonstrated clinically significant curve progression until Risser V. In females, scoliosis beyond Risser IV can be considered as an adult curve; Scoliosis in males, however, can be evaluated as an adult curve only at Risser V.

Bracing has been shown to effectively prevent curve progression in adolescent girls [5], but it is not always effective for the males [6,7]. Karol reported the result of bracing in 112 boys with AIS. 74% of these boys progressed by more than 6°, which is more than failure rate of bracing in girls. Moreover, the amount of curve correction among male patients in brace is lower compared to girls'. It has also been suggested that the spine is stiffer in males than in females [6].

Despite the importance of gender difference in curve behavior and the results of brace treatment, there are a limited number of studies comparing the results of surgical treatment between males and females. Thus, the purpose of this study was to compare the radiographic outcome of surgery for AIS between males and females in matched and unmatched groups in regard to age, curve type, and magnitude.

## Methods

Methodologically, a retrospective review of the records of all patients who had been surgically treated for AIS between May 1996 and September 2005 at our hospital was performed at first. 18 patients were treated with Harington instrumentation and 132 patients with modern segmental spinal instrumentation (Cotrel-Dubousset; CD: 24, Diapason: 103, and Universal Spine System; USS: 5 cases). The patients who were treated by only anterior surgery were excluded. Radiographic measurement were performed on standing posteroanterior and lateral radiographs of the total spine (T1-S1) acquired before surgery, at 4 days, 6 weeks, 6 months, 1, and 2 years respectively after surgery and at final follow-up. The Cobb method was used to measure the curve magnitude [8].

**Figure 1:**
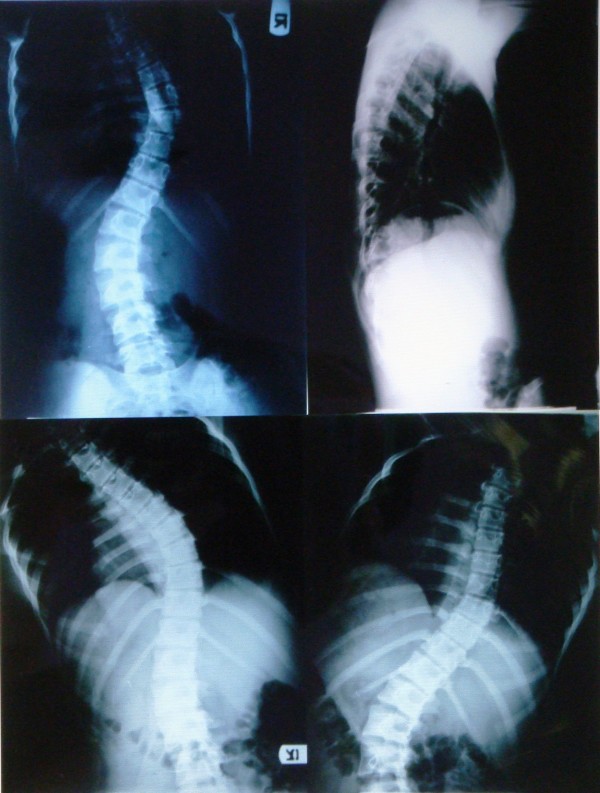
**A 15 years old boy presented with AIS.** Above: preoperative standing posteroanterior and lateral views, below: supine left and right bending films.

At the next step, preoperative coronal curve flexibility measurements from the right and left supine side bending radiographs were acquired. These views were all taken while the patients actively bent laterally (Figure 1). In order to calculate the percentage of flexibility, we subtracted the magnitude of the bend Cobb angle from the magnitude of the preoperative upright coronal Cobb angle and then divided it by the preoperative upright coronal Cobb angle calculated the percentage of flexibility. For calculating postoperative percent correction of the coronal curves, we Subtracted the magnitude of the coronal Cobb angle at final follow-up from the preoperative coronal Cobb angle and then divided it by the preoperative Cobb angle calculated postoperative percent correction of the coronal curves. The King classification was used to categorize the curve types [9].

**Figure 2:**
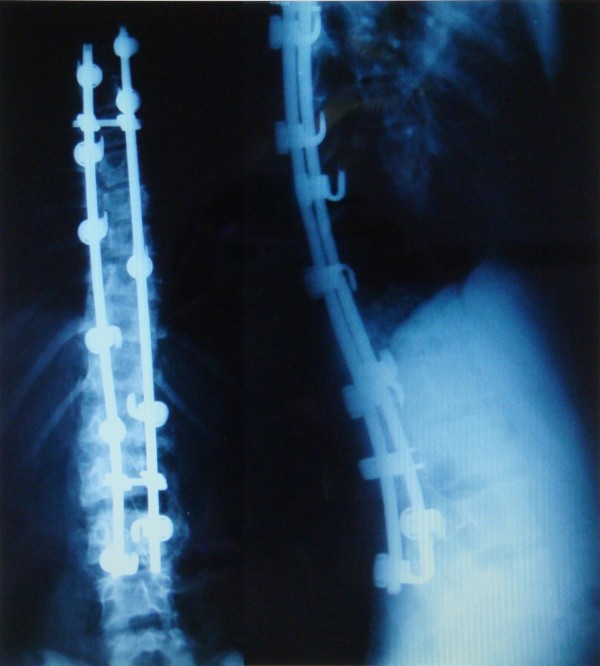
**Postoperative radiographs of the patient on Figure 1. **Posteroanterior and lateral standing views 5 years after surgery. The patient was completely asymptomatic at the latest follow-up visit.

Our threshold level for doing only PSF (posterior spinal fusion) or combined ASF (anterior spinal fusion) and PSF was a curve magnitude of 70°. We used bending views mostly for determining the fusion levels. In anterior surgery, we released the most rigid segment of the spine and then inserted autogenous (in the thoracic area and from the harvested rib) or allogenous (in the lumbar area) type of cancellous bone graft in the intervertebral spaces without implanting the spine. PSF and instrumentation with or without anterior surgery were conducted in all patients. These combined procedures were done in separate sections with the interval of 5 to 7 days. The details of the types of instrumentation used for our operative technique have been reported previously [10]. The USS was implanted according to the manufacturer's instructions [11]. PSF included decortication of the laminae, facet joint cleaning, and use of local bone graft besides an autograft from the posterior iliac crest (Figure 2).

The comparison between male and female patients was done in two stages. At first, all of the males were compared with all of the females. In the second stage, each male patient was matched with a female based on the factors such as age (± 1 year), curve type (according to the King classification), curve magnitude (± 5), and instrumentation used so that the final study series comprised 38 matched pairs.

Statistical analysis was performed by the x^2 ^test or the Mann-Whitney test. P value equal to or below 0.05 was considered statistically significant.

## Results

This analysis includes the data for 150 patients (112 females; 74.7% and 38 males; 25.3%). All patients had clinical and radiographic follow-up of at least 2 years. The mean age of the males at the time of operation was 17.3 ± 2.2 years and that of the females 16.3 ± 2.8 years (p = 0.049; significant). The mean follow-up time was 3.6 years (range 2.3 – 10.2 years).

The King classification distribution between the two gender is listed in table 1. There is similar distribution curve pattern between male and female, with King type III making up 47.4% and 47.3% of all the curve types for males and females, respectively.

**Table 1:** Curve pattern according to King classification

King Classification	Male (%)	Female (%)
I	6 (11)	11 (9.8)
II	9 (23.7)	32 (28.6)
III	18 (47.4)	53 (47.3)
IV	2 (5.2)	9 (8)
V	3 (7.9)	7 (6.3)

We performed PSF alone on 11 males (28.9%) and 58 females (51.8%). ASF and then PSF with instrumentation were conducted on the rest. Harrington rod was used in 4 (10.5%) male and 14 (12.5%) female patients and segmental spinal instrumentation in other cases.

At first, in comparing all male patients with all females, the males had greater mean age and primary curve magnitude, but less flexibility and correction percentage that were statistically significant. Loss of correction was comparable between the two groups (table 2).

**Table 2:** The results of the comparison all males with all females

	**Males**	**Females**	**P value**
**Preoperative**			
Age (year)	17.3 ± 2.3*	16.3 ± 2.9	0.049
Primary curve (°)	71.4 ± 21.3	62.7 ± 17.5	0.013
Flexibility (%)	30.1 ± 13.5	40.3 ± 17.8	0.01

**Postoperative**			
Primary curve (°)	35.3 ± 16.4	26.6 ± 15.4	0.03
Correction (%)	51.3 ± 12.9	58.8 ± 16.6	0.013

**Final visit**			
Primary curve (°)	37.6 ± 16.3	29.1 ± 15.6	0.04
Loss of correction (°)	2.3 ± 1.9	2.6 ± 2.3	0.528 (NS)

*Mean ± Standard Deviation NS: Not Significant

In the second stage of the study, the 38 male patients were compared with the 38 matched females to determine whether gender difference had an effect on the operative results (table 3). In this comparison, flexibility percent was the only index that had a statistically significant difference (the boys had more rigid curves). The correction percentage and loss of correction in boys were less than girls but these were not statistically significant (p = 0.11 and 0.25 respectively). We performed only PSF in 13 and 11 female and male patients in this group respectively. In other cases, ASF and then PSF with instrumentation were conducted. Therefore, the difference in prevalence of the type of surgery in matched group was not statistically significant, (p > 0.05).

**Table 3:** The results of the comparison between males and matched females

	**Males**	**Females**	**P value**
**Preoperative**			
Primary curve (°)	71.4 ± 21.3*	70.2 ± 18.5	0.79 (NS)
Flexibility (%)	30.1 ± 13.5	38.1 ± 17.5	0.027

**Postoperative**			
Primary curve (°)	35.3 ± 16.3	31.6 ± 18.8	0.35 (NS)
Correction (%)	51.3 ± 12.9	57.1 ± 18.2	0.11 (NS)

**Final visit**			
Primary curve (°)	37.6 ± 16.3	34.4 ± 18.9	0.35 (NS)
Loss of correction (°)	2.3 ± 1.9	2.8 ± 2.2	0.25 (NS)

* Mean ± Standard Deviation  NS: Not Significant

## Discussion

In the initial reports of segmental spinal instrumentation in the treatment of AIS, some radiographic distinctions between boys and girls had been reported [12,13]. According to these reports, between 10% and 30% of patients requiring operative intervention for AIS are males [3,14]. We found a similar percentage in our study (i.e., of 150 operative patients in this series, 25.3% were male).

A comparison of the surgical treatment outcomes in both genders has only been reported from few investigations [15-17]. We found that preoperative curve pattern between the genders are roughly similar in King classification distribution. However, the older male patients had bigger curve magnitude, and less preoperative primary curve flexibility than female patients. This observation contradicts what has been previously reported by Sucato et al [15]. They found larger primary male curves with similar curve stiffness in their comparison of male-female patients.

Marks et al. in a study of 547 (449 females and 98 males) patients, found that male AIS patients had more rigid primary curve compared to females but showed a similar degree of postoperative scoliosis correction [16]. They concluded that differences in the preoperative status and perioperative course did not compromise the outcomes of surgical treatment as in all other measures; moreover, the results were comparable between the genders.

Regardless of the preoperative differences and slight variation in treatment approaches, our study revealed that surgical outcomes are comparable between the genders. Primary curve percent correction and loss of correction over time were not statistically different between the genders.

According to our knowledge, there are only two matched studies of the surgical treatment of AIS between male and female patients:

In the first, Helenius and coauthors compared the results of operative treatment of 30 male and female AIS pairs. They finally concluded that the curves in males appear to be more rigid than in females; however, posterior surgery for AIS provides similar short and long-term results in both genders [17].

The second study that was conducted by Sucato et al. [15], revealed that treatment outcome differences did exist. They reported less correction of the curve in males compared to females. In an attempt to explain this finding, they theorized that perhaps the male patients produce a more powerful supine bend effort, reflected by a greater preoperative flexibility that the surgeon cannot duplicate at the time of surgery. Our findings about preoperative stiffer curves in males yet equal curve correction between the genders after surgery discredit this theory.

## Conclusion

In conclusion, male patients with AIS have the similar curve pattern as that of female patients. Males had more rigid primary curves compared to females but a similar degree of postoperative scoliosis correction. Male AIS patients were older at the time of surgery. These preoperative gender differences, however; did not compromise the radiological outcomes of surgical treatment and the results were comparable between the genders.

## Competing interests

The authors declare that they have no competing interests.

## Authors' contributions

EA, the senior surgeon and has made substantial contributions to conception and design of the manuscript. HB has been involved in drafting the manuscript, participated in the sequence alignment. BaM has made substantial contributions to acquisition of data from literature. FOK holds a spine fellowship. He is a junior surgeon and has had substantial role in preparing and revising the manuscript. BeM is an orthopedic surgeon and very helpful in collecting the data. He has made important critical contributions to manuscript revision in terms of its intellectual content. All authors read and approved the final manuscript.
